# Effect of access cavities on the biomechanics of mandibular molars: a finite element analysis

**DOI:** 10.1186/s12903-023-02878-3

**Published:** 2023-04-02

**Authors:** Xiao Wang, Dan Wang, Yi-rong Wang, Xiao-gang Cheng, Long-xing Ni, Wei Wang, Yu Tian

**Affiliations:** Department of Endodontics, School of Stomatology, State Key Laboratory of Military Stomatology & National Clinical Research Center for Oral Diseases & Shaanxi Clinical Research Center for Oral Diseases, The Fourth Military Medical University, 710032 Xi’an, China

**Keywords:** Cavity, Finite element method, Fracture resistance, Minimally invasive endodontics

## Abstract

**Introduction:**

This study aimed to predict the fracture resistance of a mandibular first molar (MFM) with diverse endodontic cavities using finite element analysis (FEA).

**Methods:**

Five experimental finite element models representing a natural tooth (NT) and 4 endodontically treated MFMs were generated. Treated MFM models were with a traditional endodontic cavity (TEC) and minimally invasive endodontic (MIE) cavities, including guided endodontic cavity (GEC), contracted endodontic cavity (CEC) and truss endodontic cavity (TREC). Three loads were applied, simulating a maximum bite force of 600 N (N) vertically and a normal masticatory force of 225 N vertically and laterally. The distributions of von Mises (VM) stress and maximum VM stress were calculated.

**Results:**

The maximum VM stresses of the NT model were the lowest under normal masticatory forces. In endodontically treated models, the distribution of VM stress in GEC model was the most similar to NT model. The maximum VM stresses of the GEC and CEC models under different forces were lower than those of TREC and TEC models. Under vertical loads, the maximum VM stresses of the TREC model were the highest, while under the lateral load, the maximum VM stress of the TEC model was the highest.

**Conclusion:**

The stress distribution of tooth with GEC was most like NT. Compared with TECs, GECs and CECs may better maintain fracture resistance, TRECs, however, may have a limited effect on maintenance of the tooth resistance.

## Introduction

The primary goal of endodontic treatment is the long-term retention of functional teeth but increasing the long-term retention rate of endodontically treated teeth (ETT) is still a great challenge. For decades, dental practitioners have been looking for ways to enhance fracture resistance, because tooth fracture is considered one of the main reasons resulting in the extraction of ETT [1]. Benefitting from technological advances in optics, radiology, instrumentation, materials and computer systems over the last decades, the concept of minimally invasive endodontics (MIE) to preserve as much sound dentin as possible was proposed to enhance fracture resistance [2] .

Even 10 years after the first proposed application and the religious support from proponents and influencers in the field of endodontology, MIE is still controversial because many critical aspects still remain to be studied, and no clear evidence shows that MIE is better than traditional endodontics in fracture resistance [3–5]. Some important factors may affect the fracture strength of ETT, such as structural integrity [6], morphology [7], sizes of root canal preparations [8] and prosthetic reasons [9]. Among them, structural integrity, in which marginal ridge and pericervical dentin (PCD) matter, was thought to be a crucial aspect [10]. MIE cavities are applied to conserve structural integrity by using different cavity designs in building pathways to each root canal during endodontic treatment. Several designs of MIE cavities have been proposed, including contracted endodontic cavities (CECs)[5], which are also known as “ninja” or ultraconservative endodontic cavities [11], truss endodontic cavities (TRECs) [12], and computer-aided design guided endodontic cavities (GECs) [13] .

Finite element analysis (FEA) is a promising theoretical stress analysis method because the sample demand is small and the stress inside the model can be displayed, indicating the fracture risk areas intuitively [14]. Additionally, it allows for good control of variables in experiments, overcoming some drawbacks of in vivo studies, such as bias in sample selection [5, 15].

To study whether or which MIE cavity can better maintain the resistance of tooth fracture after endodontic treatment, different MIE cavity models of a mandibular first molar (MFM) were established using a three-dimensional finite element method. A 3-point vertical/lateral static force load simulating normal masticatory force and an 8-point vertical static force load simulating maximum bite force were applied on the occlusal surface of different models. The null hypothesis is that different endodontic cavities behavior similar on stress distribution for an MFM. The maximum von Mises (VM) stress and three cross sections of different models were evaluated.

## Materials and methods

### Subjects

With consent of the patient and approval by the ethics committee of the School of Stomatology of the fourth Military Medical University (IRB-REV-2,020,044), a fresh, intact, non-carious, lightly wear, mature human MFM with three canals was obtained and scanned with a micro–computed tomographic scanner (Y. Cheetah, Germany). The scanning parameters were as follows: 80 kV, 10 W, and 1.5 μm slice thickness. The image data were exported in digital imaging and communications in medical (DICOM) format. A MIMICS 16.0 interactive medical imaging system (Materialise; Belgium) was used to identify the different hard tissues and design different endodontic cavities. Three-dimensional (3D) objects (enamel and dentin) were automatically created in the form of masks and exported as STL files. These files were refined with reverse engineering software (Geomagic Studio 10, NC). The enamel and dentin were combined using 3-Matic Research 12.0 (Materialise; Belgium).

### Cavity designs

In this research, a natural tooth (NT) and 4 endodontically treated MFM models were investigated.

**Fig. 1:**
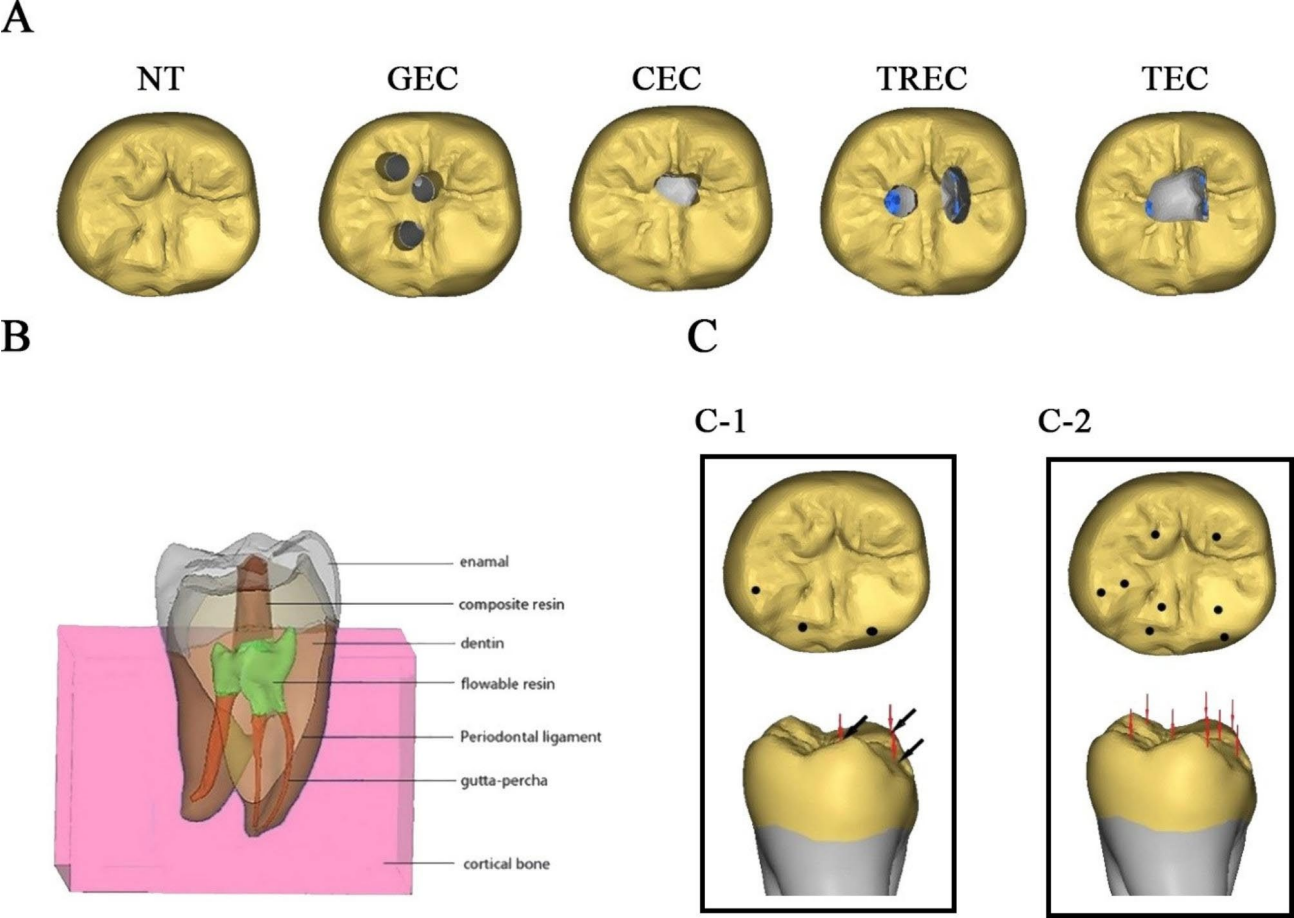
Schematic diagrams of different finite element models, components in one model and 3 force loading conditions **A.** Representative schematic diagrams of different finite element models: NT (natural tooth), GEC (guided endodontic cavity), CEC (contracted endodontic cavity), TREC (truss endodontic cavity), and TEC (traditional endodontic cavity) models **B.** Representative schematic diagram with different components in one finite element model **C.** Representative schematic diagram of different force load patterns: vertical and lateral static force loads (three contact points, 225 N in total to simulate normal vertical mastication force load) were applied on the occlusal surface (**C-1**), and vertical static force loads (eight contact points, 600 N in total to simulate the maximum bite force load) were applied on the occlusal surface. (**C-2**)

According to the volumes of removed coronal dentin and PCD, 4 cavity preparations were adopted, including the GEC [13], CEC [2], TREC [12] and traditional endodontic cavity (TEC) [14]. (Fig. 1A)

The pathways of the GEC model were three separating cylinders in the direction of the coronal 1/3 of every root canal so that the PCD could be preserved as much as possible. The CEC outline was determined with three cylinders straight into the root canal orifices meeting the occlusal surface. The outline of TREC was determined with three cylinders straight to the root canals and vertical to the partially overlapping occlusal surface.

### Geometry acquisitions

With the appropriate modifications of the microcomputed tomographic data, 3-dimensional models of endodontically treated teeth were created based on the cavities described previously. Root canal therapy was simulated by computer according to a standardized procedure [16]. The distal root canal was enlarged to 0.40 mm/0.06 taper files based on the canal geometry. The mesial root canals were enlarged to 0.25 mm/0.04 taper. The working length was set at 0.5 mm coronal to the apical foramen. The enlarged root canals were filled with gutta-percha. From the root canal orifice to the pulp chamber roof, every cavity was restored with flowable bulk resin (3 M ESPE-Filtek Bulk-Fill Flowable, USA). The rest of the cavity was restored with bulk fill composite resin (3 M ESPE-Filtek Bulk-Fill Posterior Restorative, USA). The material properties were referenced from the literature and are listed in Table 1.

**Table 1:** Mechanical Properties of the Investigated Materials [39–41]

Material	Young’s modulus (GPa)	Poisson’s ratio
enamel	84.1	0.30
dentin	18.6	0.31
pulp	0.002	0.45
periodontal ligament	0.0689	0.45
gutta-percha	0.0069	0.45
cortical bone	13.7	0.3
bulk fill composite resin	13.46	0.18
flowable bulk fill composite resin	12.0	0.25

The periodontal ligament was generated by creating a uniform 0.25 mm layer around the root [17]. Meanwhile, the alveolar bone was modelled as a 20-mm cube around the periodontal ligament. Overall, the finite element models were constituted by 7 fundamental parts (Fig. 1.B). The number of tetrahedral elements (4-tet) for the models ranged from 443,973 to 526,923. The volumes of dentin and enamel in each model were recorded.

### Model generation

The FEM was performed using HyperMesh 14.0 (Altair, USA) to calculate VM stress in the enamel and dentin. The analysis was based on the following assumptions:

1. Each material was presumed to be homogeneous, isotropic and linearly elastic.
2. There was perfect bonding between each component.
3. There was no flaw in the initial model.
4. There were rigid constraints on the base and lateral surfaces of the alveolar bone.

### Force loading processes

The force loading processes [18] were as follows:

Load A (3-point vertical force load): The models received a vertical static force load of 225 N (N) in total to simulate a normal vertical mastication force load. The force load was applied to the occlusal surface at 3 registered contact points (i.e., separately located at the mesiolingual cusp, mesiobuccal cusp and distal cusp) (Fig. 1. C-1, red arrow).

Load B (3-point lateral force load): A static force load of 225 N was applied laterally (45° to the tooth axis) at 3 registered contact points (i.e., separately located at the mesiolingual cusp, mesiobuccal cusp and distal cusp) to simulate the lateral mastication force load (Fig. 1. C-1, black arrow).

Load C (8-point vertical force load): A static force load of 600 N was applied vertically to 8 contact points registered (i.e., separately located at the mesiolingual cusp, the distolingual cusp, mesiobuccal cusp, the distobuccal cusp, and the distal cusp) on the occlusal surface to simulate the maximum bite force load (Fig. 1. C-2, red arrow).

The force load at each contact point was applied over a specified contact surface area. The maximum VM stress and VM stress in each model were computed and analyzed. The distribution of VM stress on the cross-sectional images at the level of the cemento-enamel junction (CEJ), the pulp chamber floor (PCF), and the apical foramen (AF) was investigated [14]. A convergence test was conducted to determine the size of elements in finite element modeling and the mesh was refined continuously until the FEA result did not change greatly. Mega pascal (MPa) was used as the unit of stress.

## Results

**Fig. 2:**
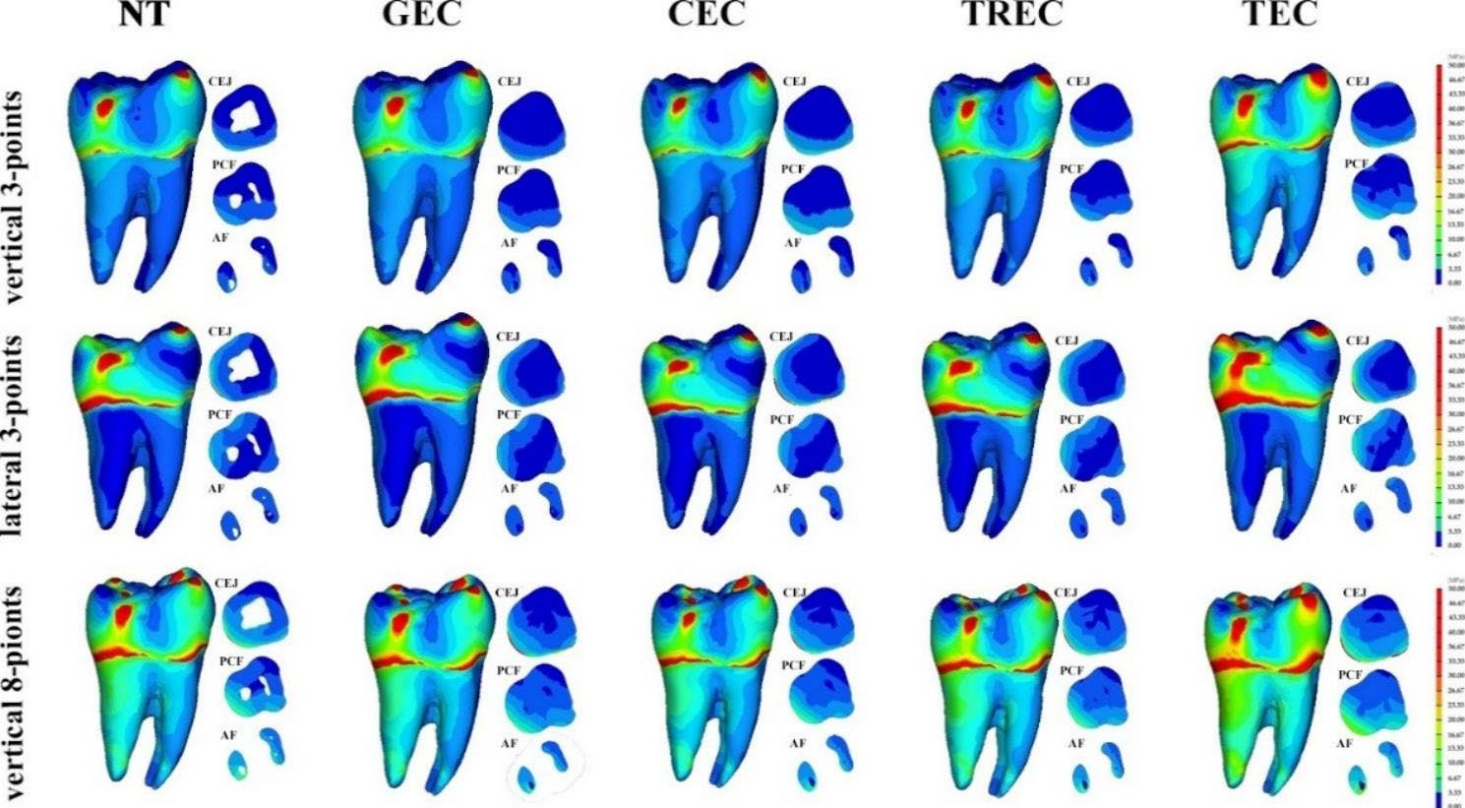
Diagrams of VM stress in different finite element models under three force loads

**Fig. 3:**
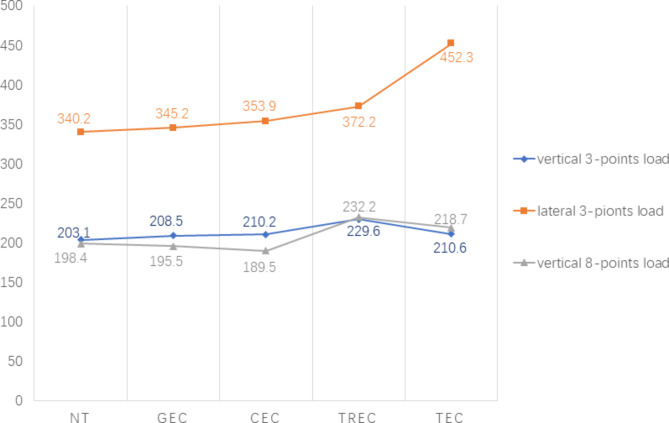
The maximum von Mises stress values (MPa) in each model

All five three-dimensional finite element models were established successfully. The VM stress diagrams of all the models are listed in Fig. 2. The VM stress value increases gradually from blue to red. In all models, the sites around the force load points and cervical regions were red, indicating higher VM stress (Fig. 2). The VM stress on the occlusal surface was spread in an approximate pattern from the force load points. The part with higher VM stress in one model is the area more prone to cracking of the model. The maximum VM stress is the peek value of VM stress in each model under different loads. Under certain stress load, the model with the highest maximum VM stress was the one most prone to fracture in all models. The maximum VM stress in all models occurred at the sites around the force load points on the occlusive surface of the model, and the values of maximum VM stresses are listed in Fig. 3.

By calculating the difference in volume cavity preparation between ETT models and NT, the volumes of removed dentin in GEC, CEC, TREC, and TEC were 33.16 mm^3^, 23.17 mm^3^, 28.34 mm^3^ and 45.57 mm^3^.

Under the vertical 3-point force load, the maximum VM stresses in the NT, GEC, CEC, TREC and TEC models were 203.1 MPa, 208.5 MPa, 210.2 MPa, 229.6 MPa and 210.6 MPa, respectively (Fig. 3). The maximum VM stress of the NT model was the lowest in all models, while that of TREC was the highest. Both the maximum VM stresses of the GEC model and CEC model were lower than those of the TREC model and TEC model. Although the maximum VM stress of the TEC model was lower than that of the TREC model, the VM stress in the investigated sections and most part of the crown in the TEC model was higher than that in the TREC model (Fig. 2). The maximum VM stress of the CEC model was slightly higher than that of the GEC model. The VM stress of the CEC model in the CEJ and PCF sections was higher than that of the GEC model, while in most parts of the crown, it was lower (Fig. 2).

Under the lateral 3-point force load, the maximum VM stresses in the NT, GEC, CEC, TREC and TEC models were 340.2 MPa, 345.2 MPa, 353.9 MPa, 372.2 MPa and 452.3 MPa, respectively (Fig. 3). The maximum VM of the NT model was the lowest, while that of the TEC model was highest in all models. The maximum VM stress of the TREC model was higher than that of the CEC model and GEC model. The maximum VM stress of the CEC model was higher than that of the GEC model. In the investigated sections, the VM stresses of the GEC and CEC were similar, but in certain parts of the crown, the VM stress of the GEC was remarkably higher (Fig. 2).

Under the vertical 8-point force load, the maximum VM stresses in the NT, GEC, CEC, TREC and TEC models were 198.4 MPa, 195.5 MPa, 189.5 MPa, 232.2 MPa and 218.7 MPa, respectively (Fig. 3). The maximum VM stress of the NT model was slightly higher than that of the CEC model and GEC model. The maximum VM stress of the TEC model was slightly lower than that of the TREC model but remarkably higher than that of the GEC model and CEC model. The maximum VM stress of the TREC model was much higher than that of the other models, while that of the CEC was the lowest. The VM stress in the crown of the NT model was slightly higher than that in the GEC model and CEC model, but in the PCF and AF sections, it was lowest in the ETT models. Although the VM stress in the zones on the occlusive surface of the TEC model was lower than that in the TREC model, the VM stress in most parts of the TEC model was the highest (Fig. 2). In the investigated sections, the VM stresses of CECs and GECs were lower than those of TRECs and TECs. The VM stress in the CEJ and PCF sections of the CEC model was higher than that of the GEC, but in the crown, it was lower (Fig. 2).

## Discussion

Since different designs of the MIE cavity have been proposed, many practitioners have already begun to apply them in clinical practice [2, 19, 20]. Previous studies have reported conflicting results regarding the influence of cavities on the fracture resistance of ETT. Many studies have shown that there are no statistically significant differences in resistance to failure between MIE cavities and TECs [21–23]. However, some studies have shown that compared with TEC, TREC and CEC improved the fracture resistance [12, 24, 25]. The MFM is the first erupting permanent tooth, and as a result, it is susceptible to decay or caries; thus, in many cases, it requires endodontic treatment [26]. It also has the highest risk of tooth fracture and is extracted after endodontic treatment [1, 27]. Therefore, an MFM was selected as the object of the study.

The straight-line access is paramount to successful shaping, allowing for easier location of the root canal, more sufficient instrument preparation, and fewer preparation complications. [28] In previous MIE cavity designs, to pursue more reduction in the size of the cavity, prognosis of root canal treatment may be compromised. [4, 23] However, a recent study shows there was a negative correlation between the amount of dentin in the coronal region and the fracture resistance of the tooth.[29] In our study, the straight-line access of GEC was simulated using cylinders in the same orientation as coronal 1/3 of every root canal. The design of GEC retains both the advantage of reducing the loss of PCD and the straight-line access, balancing biomechanical properties and clinical convenience.

In this study, different MIE cavity models were established using the three-dimensional finite element method and were compared with NT and TEC. The MIE cavity designs could reserve more hard tissue, especially PCD, compared with TEC. Since all cavity models were computer-designed, the calculated hard tissue removal was ideal. From clinical perspectives, the root canal orifices of the teeth with CECs and TRECs could not be directly located, so it was almost certain to remove more hard tissue in the root canal detection than in the in vitro model. Thus, CECs and TRECs may increase the potential for deviations and/or instrument fracture [30]. However, in GEC, the volume of tooth hard tissue removal can be controlled within a certain range, and the difference between the long axis of the root canal under TEC and GEC is acceptable [31].

The null hypothesis of this study was rejected. Under the 3-point vertical and lateral force loads, the maximum VM stress and VM stress in all investigated sections of NT were the lowest. Therefore, NT may have the best fracture resistance under normal masticatory force loading. Under 8-point vertical load, the VM stress in the crown of the NT model was slightly higher than that in the CEC model and GEC model; however, in the root, it was the lowest in all models, which may indicate that under maximum bite force load, root fracture infrequently occurs in NT. However, the incidence of crown fracture in NT may be higher than ETT with CEC and GEC.

Consistent with previous studies [14, 15], the sites around force load points and the cervical region in each model had higher VM stress, indicating that apart from the sites of the force load, PCD is another stress concentration area. Compared to the GEC and CEC models, the VM stress of the TEC model were higher, while the thickness of PCD was smaller. Hence, it is predictive that ETT with TECs seems more prone to fracture than those with CECs and GECs, especially in the cervical regions. Özyürek et al. [32] found that there was no significant difference in the fracture strengths of teeth prepared using the TEC and CEC approaches. However, they found that teeth in the CEC group had more restorable fractures than teeth in the TEC group. In this respect, it is consistent with the existing study.

Some studies have demonstrated that no obvious difference was found between CEC and TEC in fracture resistance [30, 33, 34].However, the present study indicates that CECs may have better fracture resistance than TECs. The reason may be that in this experiment, all ETT cavities were computer-designed, and PCD and dentin in the pulp chamber roof were idealized to be preserved to the largest extent. In the clinic, most practitioners prepare CECs with a combination of dentists’ experience and the results of cone bone computed tomography scans. [20] It is inevitable that more PCD and dentin in the pulp chamber roof will be removed in CECs, which will weaken the fracture resistance of ETT with CECs.

Compared to CECs, GECs seem to be more operable and predictable, because GECs allow more predictable and expeditious location with significantly less substance loss [13]. Although there were some reports about the accuracy and dentin loss of a GEC [31, 35], few reports about the effect on fracture resistance of GEC were found. In this study, the maximum VM stress and the stress distribution of the GEC model under all force loads were most like NT model. It is reasonable to believe that the fracture resistance of teeth in the GEC group is most like that in the NT model. Under two vertical loads, the VM stress of a GEC in the crown was higher than that of a CEC, but in the root, it was similar or lower. Hence, compared with CECs, GECs may increase the probability of crown fracture but reduce the risk of root fracture. This is probably because pathways of root canals were in the different directions in the GEC, the transmission of the stress was dispersed. Less stress was transmitted to the PCD and the root of the tooth, and more stress concentrated in the crown may led to more restorable fracture in teeth with GECs than in those with CECs. In this respect, GECs may be superior to CECs for the long-term retention of MFMs. However, this still needs to be confirmed by further studies.

Compared to TECs, TRECs seem to have slight advantage in fracture resistance under lateral force loads, but under the vertical force load, the result seems to be the opposite. Compared to TECs, TRECs may have a limited effect on tooth resistance enhancement. Thus, the maximum VM stresses of TREC model under all forces were higher than those of GEC and CEC models. A recent study showed that teeth with TRECs have better fracture strengths than those with CECs [25]. However, the present study shows that more solid evidence is still needed to prove that TRECs have a positive effect on fracture resistance enhancement than TECs.

The result of an experimental stress analysis by Silva et al. [36] using a universal testing machine appears at first sight to contradict the present study. In fact, the load pattern in Silva’s experiment is more similar to the 8-point vertical loads in our study. In our present study, there were the other two representative loads were applied. The advantage of the MIE cavity over the TEC in terms of fracture resistance under vertical loads was also less obvious. (Fig. 3). However, under lateral load, GECs and CECs have a more obvious advantage over TECs. In this respect, the results of this experiment are in agreement with the results of the existing study.

Compared to experimental stress analysis (ESA) using extracted natural teeth, FEA allows for better control of variables and excludes bias due the experimental manipulation such as practitioner experience and variations between teeth. Therefore, the use of FEA rather than ESA has been advocated in the methodological design of studies on the effect of MIE cavity on the fracture resistance of ETT. [37]However, FEA still cannot fully simulate the real oral environment. Each occlusion cycle is characterized by complex transient loads.[38] Although different cavities were compared under the analytical conditions of the present study, only three representative loads were performed on the same tooth model in our study. [29] More appropriate loads and samples are still needed to make the experiment more convincing for further research, and more in vitro and clinical randomized controlled trials are still needed to verify the results of this study.

## Conclusion

Within the limitations of this study, the cervical region was a stress concentration area in all models. The stress distribution of tooth with GEC was most like NT. Compared with TECs, GECs and CECs may better maintain fracture resistance, TRECs, however, may have a limited effect on maintenance of the tooth resistance. GECs may be superior to CECs for MFMs in that GECs may lead to more restorable fractures.

## Data Availability

All data generated or analysed during this study are included in this published article.

## Abbreviations

MFM: Mandibular first molar
FEA: Finite element analysis
ETT: Endodontically treated teeth
NT: Natural tooth
TEC: Traditional endodontic cavity
MIE: Minimally invasive endodontic
GEC: Guided endodontic cavity
CEC: Contracted endodontic cavity
TREC: Truss endodontic cavity
PCD: Pericervical dentin
VM: Von Mises
ESA: Experimental stress analysis
